# Dynamic graph attention network based on multi-scale frequency domain features for motion imagery decoding in hemiplegic patients

**DOI:** 10.3389/fnins.2024.1493264

**Published:** 2024-11-29

**Authors:** Yinan Wang, Lizhou Gong, Yang Zhao, Yewei Yu, Hanxu Liu, Xiao Yang

**Affiliations:** ^1^School of Automation Engineering, University of Electronic Science and Technology of China, Chengdu, China; ^2^Global R&D Center, China FAW Corporation Limited, Changchun, China; ^3^Department of Orthopedics, Sichuan Provincial People’s Hospital, University of Electronic Science and Technology of China, Chengdu, China

**Keywords:** brain-computer interfaces, motor imagery decoding, dynamic graph attention network, feature visualization, stroke rehabilitaiton

## Abstract

Brain-computer interfaces (BCIs) establish a direct communication pathway between the brain and external devices and have been widely applied in upper limb rehabilitation for hemiplegic patients. However, significant individual variability in motor imagery electroencephalogram (MI-EEG) signals leads to poor generalization performance of MI-based BCI decoding methods to new patients. This paper proposes a Multi-scale Frequency domain Feature-based Dynamic graph Attention Network (MFF-DANet) for upper limb MI decoding in hemiplegic patients. MFF-DANet employs convolutional kernels of various scales to extract feature information across multiple frequency bands, followed by a channel attention-based average pooling operation to retain the most critical frequency domain features. Additionally, MFF-DANet integrates a graph attention convolutional network to capture spatial topological features across different electrode channels, utilizing electrode positions as prior knowledge to construct and update the graph adjacency matrix. We validated the performance of MFF-DANet on the public PhysioNet dataset, achieving optimal decoding accuracies of 61.6% for within-subject case and 52.7% for cross-subject case. t-Distributed Stochastic Neighbor Embedding (t-SNE) visualization of the features demonstrates the effectiveness of each designed module, and visualization of the adjacency matrix indicates that the extracted spatial topological features have physiological interpretability.

## 1 Introduction

Brain-computer interfaces (BCIs) establish a direct communication pathway between the brain and external devices and have been widely applied in the field of medical rehabilitation in recent years (Mane et al., 2020). For hemiplegic patients, BCIs can capture motor intentions from their electroencephalogram (EEG) signals, allowing them to control exoskeleton robots to perform corresponding rehabilitation movements. This method not only enhances patient engagement but also promotes neural plasticity, leading to significantly better rehabilitation outcomes compared to conventional methods (Dobkin, 2007). The motor imagery (MI) paradigm, known for its ability to generate motor intentions without external stimuli, is frequently used with exoskeleton robots to assist hemiplegic patients in upper limb rehabilitation. Consequently, motor imagery-based BCIs (MI-BCIs) have become a research focus in recent years (Park et al., 2012; Hwang et al., 2009; Ang et al., 2008). The core of MI-BCIs lies in the decoding of EEG signals. Designing decoding methods that are both highly accurate and robust has become a key area of interest within the BCI field.

The development of MI-EEG decoding methods has transitioned from traditional machine learning-based methods to deep learning techniques (Al-Saegh et al., 2021). In earlier studies, spatial features combined with traditional machine learning methods achieved promising results in decoding MI-EEG tasks. Chen et al. (2014) utilized the Common Spatial Pattern (CSP) method to extract spatial features from EEG signals and employed Linear Discriminant Analysis (LDA) to decode upper limb motor imagery tasks, achieving a binary classification accuracy of 91.25% on the BCI Competition IV-2a dataset (BCICIV2A). Wang et al. (2019) proposed a multiple patterns of MI decoding method which was based on CSP method to control virtual automatic car. The method extended the traditional binary classification of MI to multiple classification, achieving an accuracy of 75.0%. Ang et al. (2008) applied filtering algorithms to decompose MI-EEG signals into multiple sub-band signals. They then used the CSP method along with mutual information entropy to select spatial features from each sub-band signal, achieving a binary classification accuracy of 90.3% on the BCICIV2A dataset. Based on Ang’s work, Wang et al. (2020a) fuse one-versus-the-rest filter-bank common spatial pattern (OVR-FBCSP) and brain functional connectivity and to improve the robustness of classification, achieving a triple classification accuracy of 83.81%.

Due to the ability to automatically extract task-relevant underlying features from data, deep learning methods have been rapidly adopted for MI-EEG decoding (Yang et al., 2015; Sakhavi et al., 2018; Wang et al., 2020b; Zhao et al., 2019), yielding impressive decoding performance. Yang et al. (2015) arranged the spatial features extracted from each sub-band using the CSP method into a two-dimensional feature matrix. This matrix was then further processed using Convolutional Neural Networks (CNNs) to extract relevant EEG features, achieving a four-class classification accuracy of 69.27% on the BCICIV2A dataset. Sakhavi et al. (2018) introduced a novel time-domain representation of EEG signals using the Hilbert transform, which was then fed into both temporal and spatial CNNs, achieving a four-class classification accuracy of 78.78% on the BCICIV2A dataset. Wang et al. (2020b) employed the F-score to select these optimized features extracted by FBCSP method, and fed the features to the spiking neural networks (SNN) for classification, achieving a four-class classification accuracy of 81.33% on the BCICIV2A dataset. With the advancement of Brain Connectomics, researchers have discovered that the high-level brain activities, such as MI, are generated by the activation and communication between highly specialized brain regions (Bazinet et al., 2023). These topological structural features are difficult to capture using CNNs based on Euclidean distances, leading to the increasing application of Graph Convolutional Networks (GCNs) in MI-EEG feature extraction.

GCNs leverage the input topological structures (also called adjacency matrices) to converge with fewer layers and less training, and their topological structure can model the connectivity characteristics of different brain regions (Andac et al., 2021), enhancing both the performance and interpretability of the MI-EEG decoding method. Feng et al. (2021) constructed the adjacency matrix using four graph theory features, followed by the application of GCNs and CNNs to extract spatiotemporal topological features from MI-EEG data, achieving a decoding accuracy of 92.81% for fine motor intentions of upper limb movements. Wang et al. (2020c) introduced a new attention-based multiscale CNN framework to dynamical GCN model. The adjacency matrix is adaptively determined in a data-driven way to exploit the intrinsic relationship between channels effectively, with a high binary accuracy of 95.65%. Hou et al. (2022) built an adjacency matrix from EEG’s absolute Pearson matrix and utilized a GCN framework, achieving a four-class classification accuracy of 96.24% on the High-Gamma upper limb MI dataset.

In previous works, the adjacency matrices were often pre-constructed using prior knowledge and remained unchanged during training. However, the brain activation patterns and frequencies associated with MI vary among different subjects. This discrepancy makes it challenging for adjacency matrices built on prior knowledge to adapt to new subjects, significantly affecting the generalization ability of MI-EEG decoding methods. Zhang and Huang (2019) proposed a blind GCN that updates the adjacency matrix iteratively during training using a specially designed loss function, thereby generating an adjacency matrix that fully adapts to the EEG data. This approach can enhance the generalization ability of MI-EEG decoding methods to new subjects to some extent. However, due to the low signal-to-noise ratio (SNR) of EEG signals and the high noise content, the construction and iteration process of the adjacency matrix may inadvertently optimize away some critical nodes and connections, ultimately affecting method convergence and decoding performance.

To address the mentioned issues, this paper proposes a dynamic graph attention network based on multi-scale frequency domain features (MFF-DANet) for decoding upper limb MI in hemiplegic patients. The proposed method includes a multi-scale frequency domain feature extraction module, which uses 1-D channel convolutions of different scales to extract multi-scale frequency domain features. Subsequently, a graph attention convolutional network is introduced to extract the spatial topological features between different electrode channels. The electrode channel positions are used as prior knowledge to construct a graph update layer, ensuring that key nodes and connections are preserved during iteration while maintaining model interpretability. The final goal is to decode upper limb MI intentions. The contributions of this paper are as follows:

A dynamic graph attention network based on multi-scale frequency domain features (MFF-DANet) is proposed for MI-EEG decoding. It uses temporal convolutional kernels of different scales to extract multi-scale frequency domain features, followed by the introduction of a dynamic graph attention convolutional network to extract spatial topological features between different electrode channels.

The designed dynamic graph attention convolutional network uses electrode channel positions as prior knowledge to construct a graph update layer, ensuring the retention of important nodes during the iteration of the adjacency matrix.

The proposed method was validated on the PhysioNet dataset. Experimental results show that MFF-DANet outperforms other methods in both within-subject and cross-subject scenarios. t-Distributed Stochastic Neighbor Embedding (t-SNE) feature visualization analysis indicates the effectiveness of the proposed modules, and adjacency matrix visualization analysis demonstrates that the extracted spatial topological features have physiological interpretability.

## 2 Methods

This section primarily introduces the proposed dynamic graph attention network based on multi-scale frequency domain features. Section 2.1 describes the overall framework of MFF-DANet. Sections 2.2 and 2.3 detail the proposed multi-scale frequency domain feature extraction module and the dynamic graph attention convolution module, respectively.

### 2.1 Overview

**Figure 1:**
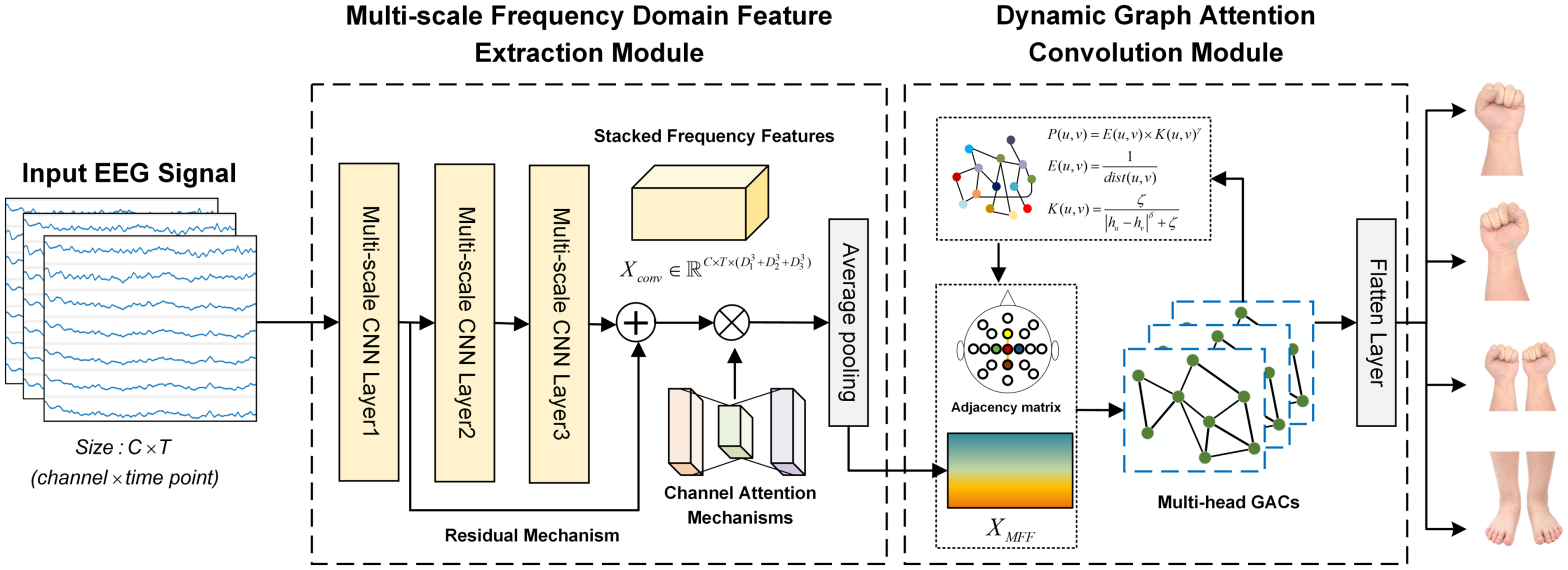
The overall framework of MFF-DANet.

MFF-DANet primarily consists of two modules: a multi-scale frequency domain feature extraction (MFF) module and a dynamic graph attention convolution (DGACN) module. The overall framework of the model is illustrated in Figure 1. The MFF module uses temporal convolutional kernels of different scales to extract frequency domain features of EEG data at various scales. These multi-scale frequency domain features are then weighted and aggregated through an average pooling operation based on a channel attention mechanism. The DGACN module introduces a graph attention convolutional network to extract and integrate the spatial topological features of different feature channels. Following this, a graph update layer is designed, combining node connection probabilities with the feature similarity to achieve dynamic updating of the adjacency matrix. This layer incorporates the spatial structure of electrode channels as prior knowledge into the initial adjacency matrix, ensuring that key node connections related to MI are preserved.

The input to the MFF-DANet framework is a set of EEG data segments $X=\left\{x_{1},x_{2},\cdots ,x_{N}\right\}$, where N is the number of samples. For each segment $x_{i}\in {\mathbb{R}}^{C\times T}$, C is the number of EEG electrode channels, and T is the number of time points. $Y=\left\{y_{1},y_{2},\cdots ,y_{N}\right\}$ represents the labels of the MI tasks, with $y_{i}\in \left\{1,2,3,4\right\}$.

### 2.2 Multi-scale frequency domain feature extraction module

**Figure 2:**
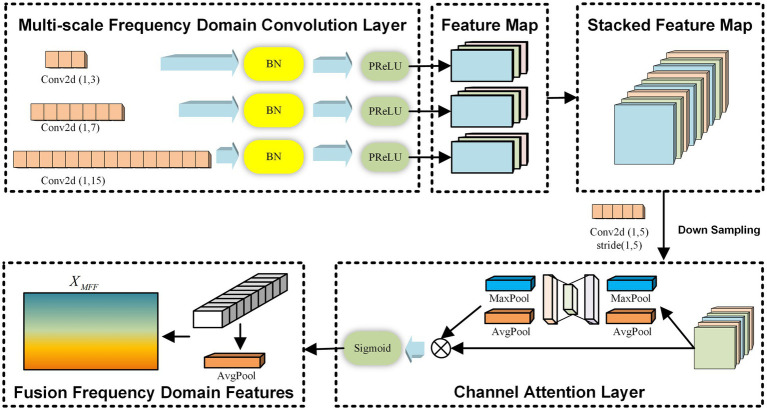
The multi-scale frequency domain feature extraction module.

The MFF module is inspired by the concept of frequency-domain convolution kernels introduced in EEGNet (Lawhern et al., 2018). In this module, three convolution kernels of different scales are designed to extract feature information from multiple frequency bands independently across channels. After obtaining the frequency domain features at different scales, an average pooling operation based on the channel attention mechanism is introduced to fuse the multiple frequency domain features, ensuring the retention of the most important frequency domain characteristics. Throughout the multi-scale frequency domain feature extraction process, the independence between electrode channels is maintained. The overall framework of this module is illustrated in Figure 2.

For the input Χ, the MFF module employs three 1D convolutional networks with different scales, each consisting of three layers. The parameters for the convolutional networks with different scales are set as $\left(1,k_{j},\;D_{j}^{l}\right)$, where $\left(1,k_{j}\right)$ represents the size of the convolutional kernel for the j-th scale ($j\in \left\{1,2,3\right\}$), and $D_{j}^{l}$ denotes the depth of the l-th layer of the j-th convolutional kernel ($l\in \left\{1,2,3\right\}$). Assuming a raw EEG signal sampling rate of 160 Hz, a convolution with $k_{j}=40$ can capture frequency information at 4 Hz and above. By setting three different kernel sizes in this way, we can simulate three overlapping high-frequency bands, effectively enabling multi-scale frequency domain feature extraction. Same Padding is applied in each convolutional network layer to ensure that the dimensions of the learned frequency domain features across different scales remain consistent. Additionally, a residual mechanism is introduced, connecting the output of the first convolutional network layer to the point just before the activation function of the third layer. This mechanism facilitates the learning of useful frequency domain features by the module. The process is described by Equations 1, 2.


(1) $$H_{j}^{l}=\sigma \left(H_{j}^{l-1}W_{j}^{l}+b_{j}^{l}\right)$$

(2) $$H_{j}^{3^{\prime }}=H_{j}^{3}+H_{j}^{1}$$

Where $H_{j}^{l}\in {\mathbb{R}}^{C\times T\times D_{j}^{l}}$ represents the output of the l-th convolutional layer at the j-th scale, with $H_{j}^{0}=X$ being the initial input. $W_{j}^{l}$ denotes the kernel weight matrix of the l-th convolutional layer at the j-th scale, and $b_{j}^{l}$ is the bias term for the same layer and scale. The symbol σ represents the activation function. The output after adding the residual connection is denoted by $H_{j}^{3^{\prime }}\in {\mathbb{R}}^{C\times T\times D_{j}^{3}}$.

The multi-scale frequency domain feature extraction module stacks the convolutional outputs $H_{j}^{3^{\prime }}$ from each scale along the feature dimension. Following this, an average pooling operation based on a channel attention mechanism is introduced to fuse the multiple frequency domain features, thereby retaining the most important frequency domain features, as described by Equations 3–5.


(3) $$X_{\text{conv}}=\text{Concat}\left(H_{1}^{3^{\prime }},\;H_{2}^{3^{\prime }},\;H_{3}^{3^{\prime }}\right)$$

(4) $$W_{att}=\text{Softmax}\left(MLP\left(X_{\text{conv}}\right)\right)$$

(5) $$X_{MFF}=\text{AveragePool}\left(X_{\text{conv}}W_{att}\right)$$

Where $X_{\text{conv}}\in {\mathbb{R}}^{C\times T\times \left(D_{1}^{3}+D_{2}^{3}+D_{3}^{3}\right)}$ represents the stacked frequency domain features. $W_{att}\in {\mathbb{R}}^{C\times T\times 1}$ denotes the channel attention weight matrix, and $X_{MFF}\in {\mathbb{R}}^{C\times T}$ represents the important frequency domain features retained by the MFF module.

### 2.3 Dynamic graph attention convolution module

The DGACN module introduces a graph attention convolutional neural network to extract spatial topological features between different electrode channels. Following this, a graph update layer is designed, combining node connection with the feature similarity to dynamically update the adjacency matrix. The graph attention convolutional network leverages a shared attention mechanism to focus on important electrode channels, making it more suitable for dynamic graph structures compared to GCNs that use spectral graph convolutions.

The core of the graph attention convolutional network is the adjacency matrix, which determines the direction of feature flow between different graph nodes. Considering the specificity of brain activation patterns during MI, we use electrode channels as nodes and the Euclidean distance between channels as the node relationship to construct the initial graph adjacency matrix E as prior knowledge. The process can be described by Equation 6.


(6) $$E\left(u,v\right)=\frac{1}{\text{dist}\left(u,v\right)}$$

Where $\text{dist}\left(u,v\right)$ represents the Euclidean distance between electrode channels u and v. The adjacency matrix element $E\left(u,v\right)\in {\mathbb{R}}^{C\times C}$ corresponds to the relationship between these channels within the constructed initial graph adjacency matrix.

Subsequently, we input the initial graph adjacency matrix and the important frequency domain features $X_{MFF}$ into a three-layer multi-head graph attention convolutional network to extract spatial topological features. Due to the low SNR of EEG signals, variations in the physiological and psychological states of subjects can easily lead to changes in brain activation patterns, and there is significant individual variability in MI-EEG signals. A fixed graph adjacency matrix struggles to adapt to this variability, so we designed a graph update layer based on the graph attention convolutional network. The graph update layer updates the probability of forming edges between nodes based on the Euclidean distance between nodes and the similarity of their topological features. This allows the graph adjacency matrix to be dynamically updated in real-time during model training using the data. This process can be described by Equations 7, 8.


(7) $$P\left(u,v\right)=E\left(u,v\right)\times K{\left(u,v\right)}^{\gamma }$$

(8) Kuv=ξ|Gu3−Gv3|Aδ+ξ

Where $P\left(u,v\right)\in {\mathbb{R}}^{C\times C}$ represents the updated adjacency matrix. $E\left(u,v\right)$ is the prior information based on Euclidean distance, indicating the inverse of the Euclidean distance between nodes u and v. $K\left(u,v\right)$ represents the topological feature similarity between nodes u and v, where $G_{u}^{3}$ and $G_{v}^{3}$ denote the spatial topological features extracted by the third layer of the graph attention convolutional network. The parameters γ, δ, and ξ are hyperparameters used to calculate the feature similarity. The graph update layer automatically updates the graph adjacency matrix during model training by incorporating both prior knowledge and sample MI-EEG data. This allows the method to reduce the impact of an initially unsuitable topological structure. Additionally, this graph update structure can uncover deeper relationships between features within the MI-EEG data and reconstruct the graph adjacency matrix, providing a certain level of interpretability. In the case of one single subject, the graph update layer can prevent false connections in the adjacency matrix caused by environmental noise or changes in the subject’s physiological or psychological state, thereby preserving the subject-specific topological feature. In the case of multiple subjects, the graph update layer can learn the shared space connectivity patterns across different subjects, which enhances our model’s ability to generalize across various subjects.

In the graph attention convolutional network, the graph attention mechanism in each layer performs a weighted summation of the updated adjacency matrix $P\left(u,v\right)$ to update the feature representation of each node. For each attention head q, the computation of graph attention convolution in each layer can be expressed by Equation 9:


(9) $$G_{u}^{l+1,q}=\sigma \left(\sum_{v\in Ν\left(u\right)}\alpha_{uv}^{q}W^{q}G_{v}^{l}\right)$$

Where $G_{u}^{l+1,q}$ represents the spatial topological feature representation of node u in the l+1-th layer for attention head q, and $G_{v}^{l}$ is the spatial topological feature representation of node v in the l-th layer. $W^{q}$ is the weight matrix for attention head q, and $\alpha_{uv}^{q}$ denotes the attention weight between nodes u and v. The output from multiple attention heads can be fused by concatenation to obtain the final spatial topological feature representation, as shown in Equation 10.


(10) $$G_{u}^{l+1}=\text{Concat}\left(G_{u}^{l+1,1},G_{u}^{l+1,2},\cdots ,G_{u}^{l+1,Q}\right)W^{O}$$

Where Q is the number of attention heads, and $W^{O}$ is the weight matrix for the output transformation.

After obtaining the final spatial topological features for each node, MFF-DANet flattens the features of all nodes and then uses a Softmax layer to map the features for classification, ultimately determining the subject’s motor intention.

## 3 Experiments

To validate the effectiveness of the proposed method, we designed a validation experiment on the public upper limb MI dataset, PhysioNet (Gerwin et al., 2004; Goldberger et al., 2000), and compared its performance with several mainstream MI-EEG decoding methods. This section mainly introduces the PhysioNet dataset, the comparison methods, and the model parameter settings.

### 3.1 Dataset description

The public dataset PhysioNet contains over 1,500 64-channel MI-EEG recordings from 109 subjects. Each subject performed four MI tasks: opening and closing the left fist, opening and closing the right fist, opening and closing both fists, and opening and closing both feet. Each MI task includes 84 trials, with each trial lasting for 4 s. The MI-EEG data in the PhysioNet dataset were recorded using equipment with a sampling rate of 160 Hz, and the data were band-pass filtered between 1–35 Hz. Due to significant missing data issues in the MI-EEG recordings for subjects #88, #89, #92, #100, and #104, these subjects’ data were excluded in this paper. Therefore, the PhysioNet dataset includes a total of 105 subjects.

### 3.2 Experimental parameter settings

#### 3.2.1 Data preprocessing

Considering the need for low-latency control in BCIs and to increase the sample size of the dataset, a sliding window operation was applied to the PhysioNet dataset. Each window has a length of 160 sampling points with a stride of 20 sampling points (Shi et al., 2024), resulting in 25 sliding windows per trial. Each subject performs four MI tasks, with 84 trials per task. Each trial’s data is segmented into 25 data fragments. Therefore, each subject in the PhysioNet dataset includes 2,100 valid MI data fragments.

#### 3.2.2 Comparison methods

The traditional machine learning-based EEG decoding method used for comparison are the widely utilized Filter Bank Common Spatial Pattern (FBCSP) (Ang et al., 2008), with Gaussian kernel-based Support Vector Machine (RBSVM) and Random Forest (RF) as classifiers. For deep learning-based EEG decoding methods, we selected EEGNet (Lawhern et al., 2018) and MCSNet (Shi et al., 2021). EEGNet is a compact CNNs framework specifically designed for EEG decoding, known for its strong generalization ability and high accuracy across various BCI paradigms. MCSNet is a physiological signal decoding method designed with a channel collaboration mechanism, demonstrating good performance in cross-subject case. Additionally, we included EEG-GAT (Demir et al., 2022), a graph convolutional neural network framework that uses a multi-head attention mechanism to parameterize the adjacency matrix, improving the model’s generalization capability for new subjects.

#### 3.2.3 Evaluation method

To thoroughly validate the effectiveness of the proposed method, we compared the decoding performance of these methods on the PhysioNet dataset under two cases: within-subject and cross-subject. Decoding accuracy is used as the performance evaluation metric. In within-subject case, all trials from one subject were divided into a training set and a test set at an 8:2 ratio, followed by a sliding window operation. The decoding accuracy on the test set is recorded as the accuracy of the decoding method. In cross-subject case, MI-EEG data from 20 randomly selected subjects from the PhysioNet dataset were used as the training set, and data from 5 randomly selected subjects were used as the test set. In both cases, the division of training and test sets was randomly repeated five times to eliminate random effects in the experimental results (Shi et al., 2024).

#### 3.2.4 Parameter settings

Based on the description of the PhysioNet dataset, C was set to 64 and T was 160. The grid search method was used to find the optimal parameters. Finally, $k_{1}$, $k_{2}$, and $k_{3}$ were set to 3, 7, and 15, respectively. $D_{1}=D_{2}=D_{3}=3$. *γ*, *δ*, and *ξ* was set to 1, 2, and 0.5, respectively. All methods were implemented using Python, with the environment configured as Python 3.8.13 and CUDA 11.7. The Adam optimizer provided by PyTorch was used during training, with a learning rate set at 0.01 and a learning rate decay set at 0.0001. The model was trained for 800 epochs. The same initial graph adjacency matrix was used for both MFF-DANet and EEG-GAT methods. The PhysioNet system follows the 64-channel international 10–10 system, with the distances between nodes determined by the electrode coordinates in the MNI head model (Valer et al., 2007). Additionally, the graph update layer was configured to update the adjacency matrix every 10 training epochs.

## 4 Results and analysis

In this section, we primarily present the MI task decoding performance of all methods under both within-subject and cross-subject cases, followed by an analysis and discussion of the results. Additionally, we utilized t-Distributed Stochastic Neighbor Embedding (t-SNE) method and the BrainNet Viewer toolbox to visualize the extracted features, aiming to validate the effectiveness of the proposed modules.

### 4.1 Experimental results in within-subject case

The decoding performance of the methods in the within-subject case reflects their learning ability with small sample data, specifically the capability to extract individualized EEG features for each subject. Table 1 presents the MI classification accuracy of each method on individual subjects.

**Table 1:** Motion intent decoding performance of all methods in within-subject case.

Subject ID	FBCSP		EEGNet	MCSNet	EEG-GAT	MFF-DANet
	SVM	RF				
S1	**68.0%**	66.4%	53.7%	51.0%	46.2%	55.4%
S2	58.4%	55.6%	62.0%	57.2%	43.4%	**66.3%**
S3	56.1%	58.6%	63.1%	48.3%	49.3%	**65.1%**
S4	56.6%	53.1%	55.0%	51.3%	39.9%	**65.1%**
S5	49.9%	47.4%	55.1%	25.0%	39.0%	**57.1%**
S6	56.6%	53.7%	**60.1%**	54.1%	36.0%	58.6%
S7	56.4%	56.9%	**70.7%**	43.7%	40.0%	55.7%
S8	58.1%	58.7%	45.0%	47.7%	40.4%	**64.3%**
S9	54.6%	55.1%	64.9%	55.9%	42.4%	**75.0%**
S10	59.0%	**59.7%**	58.4%	43.6%	45.2%	53.4%
**Average ACC**	57.4%	56.5%	58.8%	47.8%	42.2%	**61.6%**

Bolded values indicate the decoding method corresponding to the best decoding performance of the current subject.

From Table 1, it is evident that the proposed MFF-DANet method outperforms all comparison methods, achieving an average decoding accuracy of 61.6%, which is significantly higher than the comparison methods. The performances of FBCSP-SVM and FBCSP-RF are relatively close, with average accuracies of 57.4 and 56.5%, respectively, slightly lower than EEGNet’s 58.8%. MCSNet and EEG-GAT show weaker performance, with average accuracies of 47.8 and 42.2%, respectively. Considering that MFF-DANet, FBCSP, and EEGNet all incorporate modules designed to extract EEG features from different frequency bands, this highlights the importance of frequency domain features in MI-EEG decoding. It also underscores the significance and necessity of the MFF module. The methods that achieved the highest accuracy for each subject are highlighted in black in Table 1. It is clear that MFF-DANet achieves the highest accuracy for almost all subjects, particularly excelling in subjects S2 (66.3%) and S9 (75.0%). This indicates that MFF-DANet possesses superior personalized feature extraction capabilities and stability.

### 4.2 Experimental results in cross-subject case

The decoding performance in cross-subject case reflects the method’s generalization ability to new subjects. Table 2 presents the motor intention decoding performance of all methods in cross-subject case. It is evident that the proposed MFF-DANet achieved the highest average decoding accuracy, reaching 52.7%, which is significantly better than other methods. Among deep learning-based EEG decoding methods, EEGNet achieved an average accuracy of 43.8%, MCSNet achieved 45.1%, and EEG-GAT slightly outperformed them with an accuracy of 46.6%. Traditional machine learning-based EEG decoding methods performed relatively poorly, with average accuracies of 37.2 and 38.0%, which are considerably lower than those of the deep learning-based methods.

**Table 2:** Motion intent decoding performance of all methods in cross-subject case.

Subject ID	FBCSP		EEGNet	MCSNet	EEG-GAT	MFF-DANet
	SVM	RF				
**Average ACC**	37.2%	38.0%	43.8%	45.1%	46.6%	**52.7%**

Bolded values indicate the decoding method corresponding to the best decoding performance in the cross-subject case.

MFF-DANet and EEG-GAT showed a clear advantage in average decoding accuracy in cross-subject case, suggesting that GCN are better at extracting channel topological relationships that capture more common features across subjects, thereby enhancing the generalization ability of the methods. Additionally, the comparatively poor performance of traditional machine learning-based EEG decoding methods in cross-subject case may be due to the difficulty of handcrafted features effectively handling inter-individual variability.

### 4.3 Feature interpretability analysis

In recent years, developing feature interpretable methods for deep learning networks has become an active research area and is considered a crucial component of robust method validation procedures. This ensures that classification performance is driven by relevant features rather than noise or artifacts in the data (Anh et al., 2015; Ribeiro et al., 2016; Marco et al., 2017). To validate the effectiveness of the proposed modules, we employed the t-SNE method to perform dimensionality reduction and visualization of the features extracted by each module. Additionally, we used the BrainNet Viewer toolbox (Xia et al., 2013) to visualize the adjacency matrix learned by the DGACN module to observe whether the learned channel connections have physiological interpretability.

#### 4.3.1 T-SNE feature visualization

**Figure 3:**
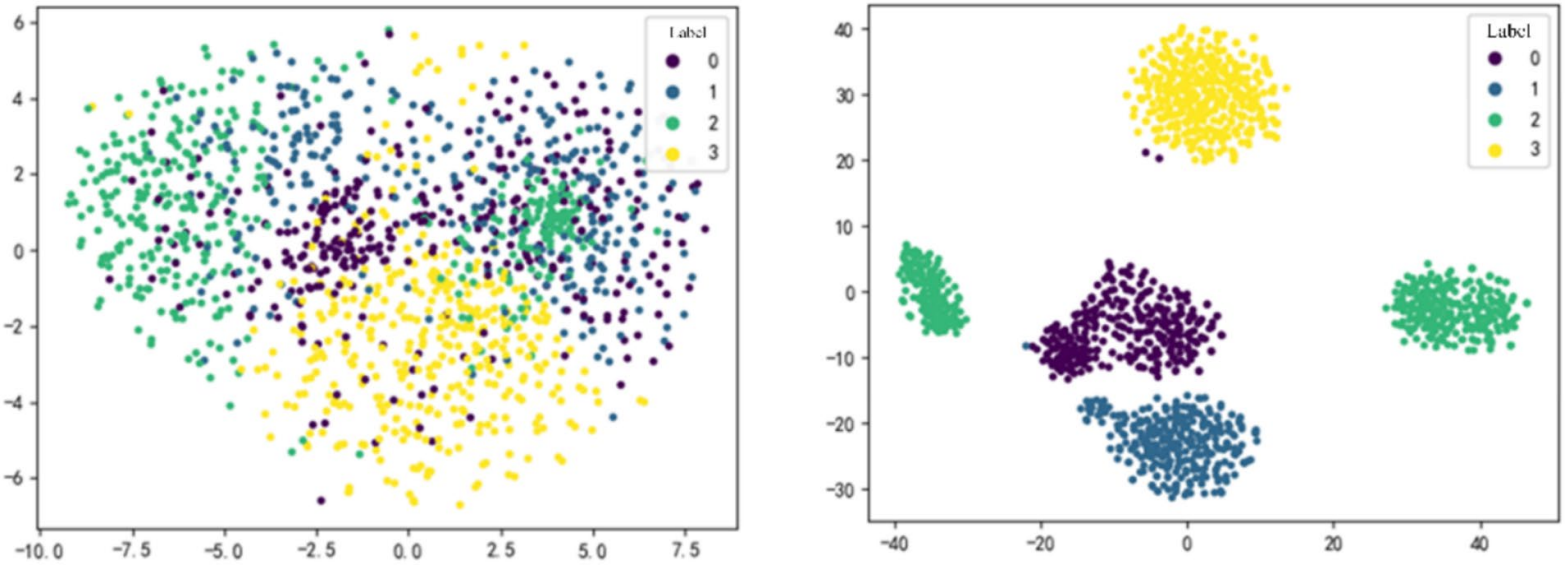
t-SNE feature visualization of MFF-DANet. The feature obtained from the MFF module (left) and the DGACN module (right).

Figure 3 shows the feature dimensionality reduction and visualization for subject S9 in within-subject case. MI-EEG data were input into the optimal model, and t-SNE method was applied to the feature maps obtained after the MFF module and the DGACN module, respectively. Different colors represent sample data with different labels, where 0, 1, 2, and 3 correspond to the four MI tasks: opening and closing the left fist, opening and closing the right fist, opening and closing both fists, and opening and closing both feet.

As shown in Figure 3, the features extracted by each module of MMF-DANet exhibit good separability, indicating that the model can capture effective frequency domain and spatial topological features from the data during training. Comparing the visualization result on the left-side and right-side, it can be observed that the separability of sample features is generally moderate after passing through the MFF module. However, the spatial topological features obtained after the DGACN module show a significant improvement in separability.

#### 4.3.2 Adjacency matrix visualization

**Figure 4:**
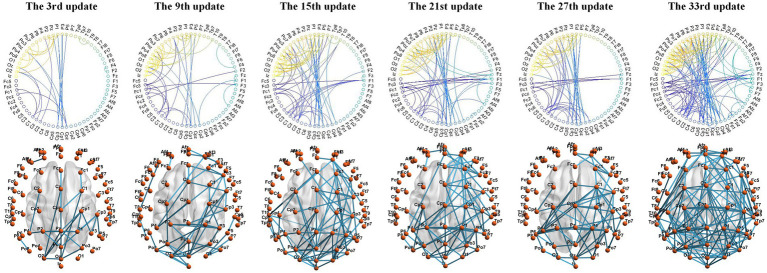
Adjacency matrix learned during MMF-DANet training iterations.

We used the BrainNet Viewer toolbox to visualize the connections between electrode channels in the adjacency matrix on a standard brain template. The MI-EEG data collected by the electrode channels are assumed to reflect the physiological electrical activity in the cortical areas projected by these electrodes. Using S9 as an example, MMF-DANet achieved the highest accuracy of 75% at the 334th epoch during training. The adjacency matrix was updated every 10 epochs, resulting in a total of 33 updates to the adjacency matrix. We visualized the adjacency matrices after the 3rd, 9th, 15th, 21st, 27th, and 33rd updates, as shown in Figure 4.

Observing the changes in the graph structure during the iteration process: at the beginning of training, there were significant differences in features between the EEG channels, resulting in fewer connections between channels after the 3rd update (30th epoch). At this stage, the Euclidean distance between electrode channels played a decisive role in the adjacency matrix. Subsequent updates revealed a more pronounced local clustering phenomenon. For example, connections were observed between electrode channels from PZ, P1, P2, P3, and P4 to CPZ, CP1, CP2, CP3, CP5, and CP6, as well as between channels from PZ, P1, P2, P3, P4, P5, POZ, PO3, PO4, O1, OZ, and O2. Additionally, connections were seen between FCZ, FC1, and FC2 to FZ, F1, and F2.

These local connections occurred between channels with relatively short Euclidean distances, mainly distributed between the sensorimotor area and the superior parietal lobule. These regions are associated with spatial orientation functions in the brain and are involved in processing visual information and sensory information from the hands. Given that the labels in the PhysioNet dataset primarily focus on upper limb MI tasks, this indicates that the DGACN module we designed effectively extracts channel topological relationships closely related to MI. The extracted features thus have physiological interpretability.

## 5 Conclusion

To address the issue of insufficient generalization ability of current MI-EEG decoding methods for new subjects, this paper proposes an MMF-DANet for decoding upper limb MI in hemiplegic patients. Given the variability in MI response frequency bands across different subjects, the proposed method utilizes convolutional kernels of various scales to extract feature information across multiple frequency bands. Subsequently, an average pooling operation based on channel attention is introduced to fuse these frequency domain features, retaining the most critical ones. Additionally, MMF-DANet incorporates a graph attention convolutional network to extract spatial topological features from different electrode channels. To ensure that the designed adjacency matrix closely aligns with the subject’s brain activation patterns during MI, electrode channel positions are used as prior knowledge to construct a graph adjacency matrix update layer. This approach ultimately enhances the decoding of upper limb MI. The proposed method was validated on the PhysioNet dataset, and the results demonstrate that MMF-DANet achieved the highest decoding accuracy in both within-subject and cross-subject cases. It effectively extracts personalized features for different subjects as well as common topological features shared across all subjects. The t-SNE dimensionality reduction visualization of the features confirmed the effectiveness of each module, while the visualization of the adjacency matrix indicated that the graph adjacency matrix update layer effectively captures spatial topological features related to MI, providing physiological interpretability.

## Data Availability

The raw data supporting the conclusions of this article will be made available by the authors, without undue reservation.
